# Prevalence of diabetes and impaired fasting glucose among residents in the Three Gorges Reservoir Region, China

**DOI:** 10.1186/1471-2458-14-1152

**Published:** 2014-11-06

**Authors:** Li Qi, Liangui Feng, Xianbin Ding, Deqiang Mao, Yulin Wang, Hongyan Xiong

**Affiliations:** Department of Military Epidemiology, College of Military Prevention, Third Military Medical University, Chongqing, China; Chongqing Municipal Center for Disease Control and Prevention, Chongqing, China; Chongqing Medical and Pharmaceutical College, Chongqing, China

## Abstract

**Background:**

The Three Gorges Dam in China is the world’s largest hydro-electric scheme in the contemporary world. After the construction of the Dam, great changes took place on the residents’ lifestyles characterized by reduced physical activity due to the loss of arable land and a series of psychological problems caused by resettlement, which might be regarded as contributing factors to the development of diabetes in Three Gorges Reservoir Region (TGRR). However, there is no study that has been conducted targeting large population samples with the aim of determining the prevalence of diabetes in TGRR. This study purposed to estimate the prevalence of diabetes and impaired fasting glucose (IFG) in the adult population ≥18 years in TGRR and to evaluate the associated risk factors.

**Methods:**

A total of 3721 randomly selected adults, aged ≥18 years and having lived in TGRR for at least one year, participated in questionnaire-based interview from April to May 2013 and had their physical examinations and standard glucose taken. 75 g oral glucose tolerance test (OGTT) was conducted on the subjects with fasting glucose levels being ≥ 5.6 mmol/L. Diabetes and IFG were defined according to WHO 1999 criteria.

**Results:**

The age-standardized prevalence of diabetes and IFG were 7.6% (7.9% among men and 7.4% among women) and 9.0% (9.1% among men and 8.9% among women), respectively. Among the identified cases of diabetes in this study, 54.46% (171/314) were newly diagnosed. The prevalence of diabetes cases rose with age (4.0%, 4.5%, 8.1%, 11.2%, 12.4% and 12.9% among persons who were 18 to 29, 30 to 39, 40 to 49, 50 to 59, 60 to 69 and ≥ 70 years of age, respectively). The results of multivariate logistic-regression analyses showed that the diabetes was significantly linked to age, family history of diabetes, central obesity, educational level and hypertension for both men and women. In addition, smoking was significantly associated with diabetes in men.

**Conclusions:**

Diabetes has become a major public health problem in the TGRR with a large number of the cases undiagnosed. These results suggest that regular population-based diabetes screening should be conducted to identify early-stage diabetes and integrated strategies aimed at the prevention and treatment of diabetes initiated.

## Background

Diabetes ranks highly on the international health agenda as a global pandemic and as a threat to human health and global economies [1]. Most people with diabetes live in low- and middle-income countries [2]. China is one of the rapidly developing middle-income countries where the prevalence of diabetes has risen rapidly over the last 30 years [3]. Data from five national surveys indicates that the prevalence of diabetes grew nearly 17-fold from 0.7% in 1980 [4] to 11.6% in 2010 [5]. Although different sampling methods, screening procedures, and diagnostic criteria were used, the data from the national surveys indicate a rapid upsurge in the prevalence of diabetes amongst the Chinese population.

The Three Gorge Dam in China is the world’s largest hydroelectric scheme in the modern-day era. The Three Gorges Dam has created a reservoir with a total water surface area of 1080 km^2^. The region surrounding the reservoir has now come to be known as the Three Gorges Reservoir Region (TGRR). Considering the unprecedented magnitude and impact of the development of TGRR, it is worthwhile to examine the health profile of residents in TGRR. Some studies have already assessed the impact of the development on the environment, economic and psychological problems and parasitic diseases and showed a substantial effect [6]. However, there is no study conducted on a large population as a sample to determine the prevalence of diabetes in the TGRR.

To understand the prevalence and relevant factors relating to diabetes in TGRR, we conducted a cross-sectional survey from April to May 2013. We aimed at compiling useful information on the health profile of residents and create a database that can be useful to the local health professionals charged with controlling and managing diabetes in the TGGR.

## Methods

### Study population

The prior presumption of the prevalence of diabetes in TGRR would be more or less similar to the rate of China in 2007 (9.7%) [7]. With 95% confidence interval, the sample size of 3,365 subjects was required for this study. The formula for the sample size is shown as follows:


Considering that the loss of follow-up rate was 10%, the total sample size was 3,701.

A multistage sampling method was used: stage 1, three regions were randomly selected from the whole of TGRR; stage 2, three towns were randomly selected from each of the sampled region; stage 3, four villages or communities were randomly selected from each of the sampled towns; stage 4, households within each village or community were listed, resulting to 110 households being randomly selected. In the final stage, 1 person at least 18 years old was randomly selected from each household using a Kish selection table [8]. When the selected individual declined or was unavailable on three occasions, a household for replacement was identified from households of similar composition in the same village or community with the exclusion of the already selected households, using a simple random sampling method. The replacements ensured an adequate sample size from each selected community or village. The inclusion criteria were that the subject be ≥18 years of age and had lived in TGRR for more than one year. However, pregnant females and mentally or physically handicapped people were disqualified from the study.

Finally, a total of 3960 people were selected and requested to participate in the survey, out of which 3721 completed and were included in the final analysis. The overall response rate was 93.96%.

The ethical clearance of this study was obtained from the ethical committee of research in Chongqing Center for Disease Control and Prevention, China. Written informed consent was obtained from each participant before data collection.

### Data collection

#### Questionnaire interview

A standardized questionnaire, designed based on the national surveillance of non-communicable diseases and its risk factors among adults in China, 2010, that covers demographic characteristics—age, gender, educational level, occupation and income, family medical history, and lifestyle risk factors —smoking, drinking, vegetable intake and physical activities, was performed by trained investigators.

Education level was categorized as ≤ primary school, junior middle school, senior middle school and ≥ College. Yearly family income was categorized as below 5,000 RMB, 5,000-9999 RMB, 10,000-49,999 RMB and above 50,000 RMB. Smoking was classified in terms of current smokers or non-smokers. Drinking was delineated as the consumption of at least 30 g of alcohol per week for 1 year or more. Vegetable intake was expressed as eating < 1 time/day, 1-2 times/day and ≥ 3 times/day. Regular physical activity was defined as participation in moderate or vigorous activity for ≥ 30 minutes/day at least 5 days per week. Static activities were delimited to sitting or lying down but without sleeping. Family history of diabetes or hypertension was defined as having at least one of the parents, brothers or sisters diagnosed with diabetes or hypertension in their lifetime.

#### Physical examinations and measurements

Height was measured twice with a tape measure attached to a wall to the nearest centimeter, whereas weight was recorded by use of a weighing scale to the nearest 0.1 kg, with the subject bare feet and wearing lightweight clothing, after which the average of the values of height and weight were worked out. Body mass index (BMI) was calculated as the ratio of weight (kg) to the square of height (m). Participants with a BMI ≥25 kg/m^2^ and <30 kg/m^2^ were classified as overweight, and those with BMI ≥ 30 kg/m^2^ were classified as obese [9]. Waist circumference was measured twice on standing participants; at the midpoint between the lower edge of the costal arch and the upper edge of the iliac crest and the mean calculated for analysis. Central obesity was defined as waist circumference of 90 cm or more in men and 80 cm or more in women [10]. Systolic blood pressure (SBP) and diastolic blood pressure (DBP) were measured three times for each subject, using a mercury sphygmobolometer while in a sitting position with an interval of 15 min rest, and the average calculated. Hypertension was defined as SBP ≥ 140 mmHg or DBP ≥ 90 mmHg, or as previously diagnosed hypertension identified by a positive response from the participant to the question, "Has a doctor ever told you that you have hypertension?"

#### Laboratory measurements

An overnight fasting venous specimen was collected in the morning using a vacuum tube containing sodium fluoride for measuring fasting plasma glucose. Plasma glucose was measured using the Hexokinase method (Toshiba TBA-40FR). All the participants with fasting plasma glucose ≥ 5.6 mmol/L and <7.0 mmol/L, expect for those with diagnosed diabetes, underwent a 75 g oral glucose tolerance test (OGTT). All control values were consistent with the standards recommended by the medical laboratory of Chongqing Center for Disease Control and Prevention.

According to the 1999 World Health Organization diagnostic criteria, diabetes is defined as the level at which fasting glucose reaches 126 mg/dl (7.0 mmol/l), or when 2 h postprandial glucose level reaches 200 mg/dl (11.1 mmol/l), or both [11]. Impaired fasting glucose (IFG) is defined as the level at which fasting glucose is ≥109 mg/dl (6.1 mmol/l) and <126 mg/dl (7.0 mmol/l). In our study, subjects were categorized into three groups: diabetes group, which included previously diagnosed diabetes and/or those currently taking antidiabetic drugs and undiagnosed diabetes, detected for the first time during the study; IFG group; normal fasting glucose group.

All investigators and staff successfully completed a two-day training program that familiarized them with the aim of the study, the specific tools and methods.

#### Statistical analysis

Prevalence estimates for diabetes and IFG were calculated for the overall population and subgroups according to age and gender. Age-standardized prevalence was calculated by way of the direct method using data from the population distribution in China, 2010.

Descriptive data was expressed as mean ± standard deviation (SD) or percentage. A Student *t-*test was used to test differences in the means of continuous variables, and the Chi-square test to test differences in ratios. The analyses were gender-specific. A multinomial logistic regression analysis, using a backward elimination method, was used to examine the association of socio-demographic, family medical history and lifestyle characteristics with the odds of diabetes. *P* <0.05 was considered to be statistically significant. All initial data was entered into Epidata software 3.1 versions and Statistical Package for Social Sciences (SPSS) 18.0 for statistical analyses.

## Results

### Demographic characteristics

Table 1 presents demographic characteristics of the subjects. Of the 3,721 participants, 1,777 (47.8%) were male and 1,944 (52.2%) were female. Their mean age (SE) was 47.39 (16.7). Significant differences were shown in marital status, education level, occupational level and family income between men and women. The percentage of central obesity and the mean of BMI for women were significantly higher than that of men, while the percentage of smoking, drinking and sedentary life for more than six hours per day of men was significantly greater than that of women. However, no significant differences were noted in terms age, family history of hypertension and diabetes, hypertension, overweight and obesity between men and women.

**Table 1:** **Characteristics of participants in Three Gorges Reservoir Region, China**

Variables	Male (n = 1777)	Female (n = 1944)	Total	X ^2^/t	***P***value
Age (%)					
18-29	319 (18.0)	328 (16.9)	647 (17.4)	3.413	0.637
30-39	328 (18.5)	334 (17.2)	662 (17.8)		
40-49	373 (21.0)	437 (22.5)	810 (21.8)		
50-59	323 (18.2)	376 (19.3)	699 (18.8)		
60-69	252 (14.2)	264 (13.6)	516 (13.9)		
>70	182 (10.2)	205 (10.5)	387 (10.4)		
Marital status (%)					
Unmarried	180 (10.1)	125 (6.4)	305 (8.2)	28.143	<0.001
Married	1490 (83.8)	1639 (84.3)	3129 (84.1)		
Widowed or divorces	107 (6.0)	180 (9.3)	287 (7.7)		
Education level (%)					
Lower than primary school	745 (41.9)	1083 (55.7)	1828 (49.1)	75.836	<0.001
Junior middle school	575 (32.4)	519 (26.7)	1094 (29.4)		
Senior middle school	320 (18.0)	252 (13.0)	572 (15.4)		
College and above	137 (7.7)	90 (4.6)	227 (6.1)		
Occupation (%)					
Peasant	936 (52.7)	1090 (56.1)	2026 (54.4)	68.183	<0.001
Laborer	396 (22.3)	252 (13.0)	648 (17.4)		
Retiree or House worker	300 (16.9)	455 (23.4)	755 (20.3)		
Others	145 (8.2)	147 (7.6)	292 (7.8)		
Income (%)					
Lower than 5000 RMB/Year	249 (14.0)	333 (17.1)	582 (15.6)	501.912	<0.001
5000-9999 RMB/year	529 (29.8)	513 (26.4)	1042 (28.0)		
10000-49999 RMB/year	216 (12.2)	158 (8.1)	374 (10.1)		
>500000 RMB/year	275 (15.5)	278 (14.3)	553 (14.9)		
Refuse to answer	508 (28.6)	662 (34.1)	1170 (31.4)		
BMI (means ± SD, kg/m^2^)	23.0 ± 3.0	23.3 ± 3.3	23.1 ± 3.2	-1.976	0.048
Waist circumference (means ± SD, cm)	78.9 ± 9.2	76.5 ± 9.2	77.6 ± 9.3	8.083	<0.001
Smoking (%)	664 (37.4)	142 (7.3)	806 (20.2)	4.940	<0.001
Drinking (%)	498 (28.0)	199(10.2)	697 (18.7)	1.930	<0.001
Vegetable intake (%)					
Less than 1 time per day	128 (7.2)	132 (6.8)	260 (7.0)	3.695	0.158
1 or 2 times per day	252(14.2)	237 (12.2)	489 (13.1)		
3 times per day	1397 (78.6)	1575 (81.0)	2972 (79.9)		
Sedentary lifestyle time per day (%)					
Less than 3 hours	1066 (60.0)	1240 (63.8)	2306 (61.9)	8.042	0.018
3-6 hours	505 (28.4)	525 (27.0)	1030 (27.7)		
More than 6 hours	206 (11.6)	181 (9.3)	387 (10.4)		
Family history of hypertension (%)	294 (16.5)	374 (19.2)	668 (18.0)	3.793	0.150
Family history of diabetes (%)	275 (15.5)	284 (14.6)	559 (15.0)	1.558	0.459
Fasting glucose (mean ± SD, mmol/L)	5.5 ± 2.5	5.5 ± 1.7	5.1 ± 2.1	442.4	0.019
Overweight (%)	466 (26.2)	527 (27.1)	988 (26.6)	0.372	0.542
Obesity (%)	101 (5.7)	127 (6.5)	215 (5.8)	1.164	0.281
Central obesity (%)	448 (25.2)	690 (35.5)	1138 (30.6)	46.238	<0.001
Hypertension (%)	524 (29.5)	572 (29.4)	1096 (29.5)	0.002	0.966

*Abbreviation*: *BMI* body mass index, *SD* standard deviation.

### Prevalence of diabetes and IFG

**Figure 1:**
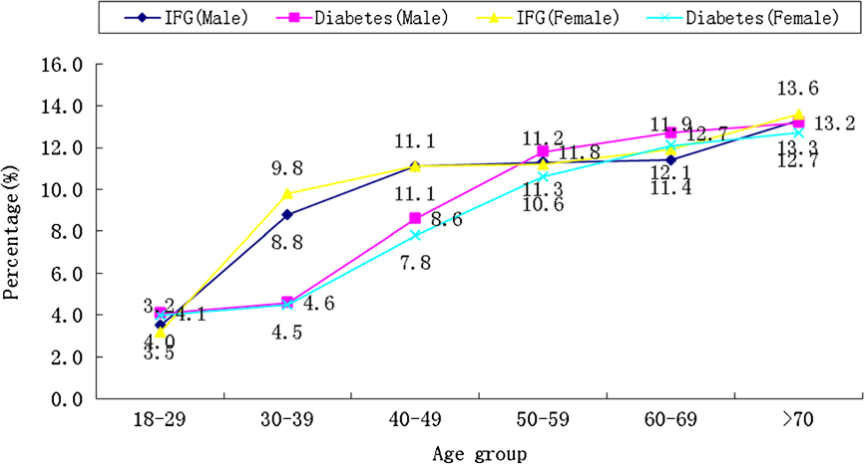
**Prevalence of diabetes and impaired fasting glucose by different age groups and gender.**

**Figure 2:**
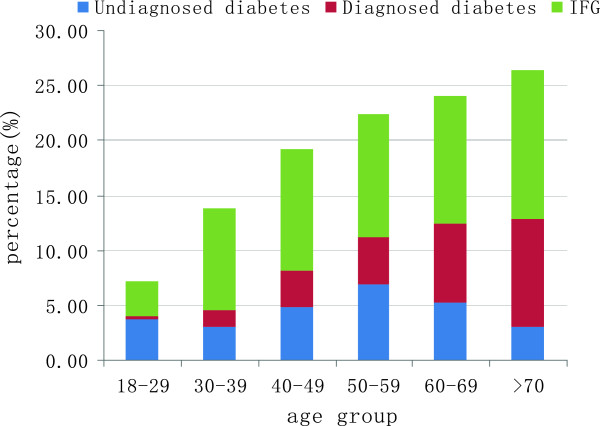
**Age-specific prevalence of diagnosed and undiagnosed diabetes and IFG among participants.**

The crude prevalence of diabetes and IFG was 8.4% (95% CI, 7.5% - 9.3%) and 9.8% (95% CI, 8.6% - 11.0%), respectively. After standardization of age, based on China’ 2010 census data, the prevalence was 7.6% (95% CI, 6.9% - 8.3%) for diabetes and 9.0% (95% CI, 7.9% -10.1%) for IFG. The age-standardized prevalence estimates of diabetes and IFG were similar for men and women (diabetes: 7.9% vs. 7.4%, X^2^ = 0.228, *p* = 0.633; IFG: 8.9% vs. 9.1%, X^2^ = 0.288, *p* = 0.592) and the prevalence of diabetes and IFG significantly increased across the age groups (*P* < 0.001) (Figure 1).Out of the identified cases of diabetes in our study, 54.5% (171/314) were undiagnosed. The ratios of undiagnosed cases to total diabetes cases were: 90.0% (24/26) in the 18-29 age group, 65.0% (20/30) in the 30-39 age group; 56.9% (40/66) in the 40-49 age group, 58.6% (48/78) in the 50-59 age group, 41.1% (27/64) in the 60-69 age group and 23.3% (12/50) in the >70 age group (Figure 2).

### Factors associated with diabetes

On univariate analysis, the following factors were significantly associated with diabetes: age, educational level, marital status, smoking, family history of diabetes obesity, central obesity, and hypertension (Table 2).

**Table 2:** **The results of univariate analysis of the potential risk factors associated with diabetes**

Variables	Total number	DM prevalence (%)	OR (95% CI)	***P***-value
Age				
18-29	647	4.0	1	
30-39	662	4.5	1.13 (0.66-1.94)	0.646
40-49	810	8.1	2.12 (1.33-3.38)	0.001
50-59	699	11.2	2.67 (1.69-4.21)	<0.001
60-69	516	12.4	2.96 (1.85-4.74)	<0.001
>70	387	12.9	3.54 (2.12-5.80)	<0.001
Educational level				
Less than junior middle school	1828	10.5	1	<0.001
≥ Junior middle school	1893	6.4	0.59 (0.47-0.72)	
Smoking				
No	2915	7.7	1	0.002
Yes	806	11.2	1.51 (1.35-1.67)	
Family history of diabetes				
No	3162	7.7	1	
Yes	559	12.6	1.71 (1.29-2.27)	<0.001
Obesity				
No	3506	8.2	1	
Yes	215	12.1	1.54 (1.00-2.36)	0.047
Central obesity				
No	2583	7.0	1	
Yes	1138	11.6	1.73 (1.37-2.19)	<0.001
Hypertension				
No	2625	6	1	
Yes	1096	14.2	2.59 (2.05-3.27)	<0.001
Marital status				
Married	3129	8	1	
Unmarried or widowed or divorces	592	11	1.39 (1.12-1.66)	0.015

*Abbreviation*: *CI* confidence intervals, *OR* odds ratio.

Multivariate logistic regression analyses showed that older age, family history of diabetes, central obesity, hypertension, and educational level below junior middle school were all significantly associated with an increased risk of diabetes for both men and women. In addition, smoking was significantly associated with an increased risk of diabetes in men (Table 3).

**Table 3:** Multivariable-adjusted odds ratios for diabetes by gender*

Variable	Male		Female	
	OR (95% CI ^&^)	***P***value	OR (95% CI ^&^)	***P***value
Age, per 10-yr increment	1.70 (1.52 - 1.89)	0.004	1.71 (1.47 - 1.95)	0.002
Less than junior middle school	1.21 (1.16 - 1.27)	0.001	1.23 (1.17 - 1.28)	<0.001
Family history of diabetes^#^	1.51 (1.27 - 1.89)	0.002	1.53 (1.41 - 1.65)	0.001
Central obesity^#^	2.15 (1.98 - 2.32)	<0.001	2.20 (2.03 - 2.38)	<0.001
Hypertension^#^	1.74 (1.56 - 1.92)	0.002	1.48 (1.31 - 1.65)	0.021
Smoking^#^	1.17 (1.04 - 1.30)	0.035	_	

*Odds ratios were calculated with the use of multinomial logit models. All covariables were included in the model.

^#^References were participants without family history of diabetes, not central obesity, not hypertension and no smoking.

^&^ *Abbreviation*: *CI* confidence intervals, *OR* odds ratio.

## Discussion

This cross-sectional study revealed that the age-standardized prevalence of diabetes among adults living in TGRR was 7.6%, which was higher than the prevalence among individuals in other cities in China, such as Guangzhou (5.5%) [12], Shanghai (6.7%) [13], Qingdao (6.1%) [14], Haikou (5.3%) [15], and Heilongjiang (7.1%) [16]. The differences between this study and other studies may be due to different environments and lifestyles of the studied subjects.

After the construction of the Three Gorge Dam, great changes took place in the residents’ lifestyles characterized by reduced physical activity due to the loss of arable land and a series of psychological problems due to resettlement, which might be regarded as contributing factors in the development of diabetes [17]. Our study is relevant as it will be useful for further research and strategies aimed at promoting healthy lifestyles in TGRR.

In our study, 54.46% of diabetes cases had been undiagnosed, which means that more than half of the patients did not know they had diabetes before. Our study was in conformity with other studies [18–22], which also found a high percentage of undiagnosed cases of diabetes. The low diagnosis rate might be attributable to the silent condition the patients undergo until the development of a complication, lack of awareness and complicated screening and diagnostic methods [18]. Compared to diagnosed cases of diabetes, undiagnosed cases are prone to various complications [19] and are likely to become a huge economic burden to society [23]. Therefore, it is essential to establish regular population-based screening to identify early-stage diabetes and offer early treatment to delay its development and reduce the related complications in TGRR.

In our study, a higher prevalence of IFG (9.0%) was recorded as compared to diabetes (7.6%). Individuals with IFG are more prone to progress to the diabetes stage in the absence of interventional measures. A previous study had reported that the progression rate from IFG to diabetes is approximately 8.8% per year [19]. The high prevalence of IFG in TGRR might be a significant risk factor in the development of diabetes. Therefore, it is necessary to conduct regular screening to identify those individuals with IFG and to provide effective interventional measures to prevent the development of diabetes.

Our study showed that the prevalence of diabetes increased with age, which indicates that aging might contribute to the diabetes epidemic among adults living in TGRR. The increase was similar to other studies in China [12] and other Asian [24], European [25] and American populations [26]. However, it was different from that of some Asian populations in which the prevalence of diabetes reached its peak in the 60-70s then declined thereafter [27]. In addition, it’s important to note that diabetes may no longer be entirely due to old age as 4.0% in 18-29 year-old group, and 4.5% in 30-39 year-old group were found to be diabetic in our study. This means that young people should also be targeted for diabetes prevention.

Similar to previous studies [3, 28–30], central obesity and hypertension were identified as risk factors for diabetes in our study. Generalized obesity is significantly associated with diabetes in univariate analysis but failed to attain statistical significance in multivariable analysis. This result could indicate that central obesity is more strongly associated with diabetes than generalized adiposity in adults living in TGRR. The prevalence of central obesity and hypertension were rather high (30.6% and 29.5%) among the study subjects. Therefore, it is essential to manage diabetes together with obesity and hypertension, especially central obesity, in order to reduce the clinical consequences such as coronary heart disease, stroke and peripheral arterial diseases.

Low educational level and family history of diabetes also appeared to be risk factors in our study. Educational level is a good indicator of socioeconomic status, with higher educational level being associated with lower levels of cardiovascular risk factors such as obesity and hypertension [31, 32]. The results of our study pointed to low educational level of residents in TGRR, with half of the participants being lower than primary school. Furthermore, the results revealed that smoking is an independent risk for diabetes in men as shown earlier [32–35]. Therefore, it is essential to conduct effective behavioral intervention programs to promote healthy ways of life in citizens, such as peer support on smoking cessation especially for people with lower education level.

This study was the first survey on diabetes and IFG among adults living in TGRR. To ensure the validity of the data collected, we employed standard instruments and protocols together with strict training. In addition, the standard laboratory methods for measuring glucose and blood pressure were used.

However, there were some limitations our study encountered. First, due to the cross-sectional nature of our study, the associations between risk factors and diabetes were not causality. Second, the investigated population was older than the general population in TGRR. Thus, the age-adjusted prevalence of diabetes was lower than the crude prevalence. Finally, 2 h OGTT was conducted only in those subjects whose fasting glucose was ≥ 5.6 mmol/L due to the financial and organizational limitations. The fasting glucose is recommended over OGTT in epidemiological studies and for individual diagnostic purposes because it is easy, convenient and acceptable to subjects, in addition to being reproducible and cost effective. However, the OGTT has the potential to identify individuals who have abnormal glucose that could not be detected by fasting glucose tests [36]. Therefore, the prevalence in our survey might underestimate the real prevalence in TGRR.

## Conclusions

In summary, our results indicate that the diabetes has become a major public health problem in TGRR. More troubling is the finding that majority of cases of diabetes are undiagnosed. Furthermore, central obesity and hypertension are highly prevalent and are strongly linked to diabetes. These findings suggest that regular population-based diabetes screening should be established to identify early-stage diabetes, and an integrated health-education program encouraged to boost public awareness of diabetes, risk factors and complications in TGRR.
